# A Quality Management Approach to Implementing Point-of-Care Technologies for HIV Diagnosis and Monitoring in Sub-Saharan Africa

**DOI:** 10.1155/2012/651927

**Published:** 2012-01-11

**Authors:** Joseph P. Shott, Ronald M. Galiwango, Steven J. Reynolds

**Affiliations:** ^1^Clinical Monitoring Research Program, SAIC-Frederick, Inc., NCI-Frederick, Frederick, MD 21702, USA; ^2^Rakai Health Sciences Program, Uganda Virus Research Institute, Kalisizo, Uganda; ^3^Laboratory of Immunoregulation, Division of Intramural Research, National Institute of Allergy and Infectious Diseases, National Institutes of Health, Bethesda, MD, USA

## Abstract

Technology advances in rapid diagnosis and clinical monitoring of human immunodeficiency virus (HIV) infection have been made in recent years, greatly benefiting those at risk of HIV infection, those needing care and treatment, and those on antiretroviral (ART) therapy in sub-Saharan Africa. However, resource-limited, geographically remote, and harsh climate regions lack uniform access to these technologies. HIV rapid diagnostic tests (RDTs) and monitoring tools, such as those for CD4 counts, as well as tests for coinfections, are being developed and have great promise in these settings to aid in patient care. Here we explore the advances in point-of-care (POC) technology in the era where portable devices are bringing the laboratory to the patient. Quality management approaches will be imperative for the successful implementation of POC testing in endemic settings to improve patient care.

## 1. Introduction

Human immunodeficiency virus (HIV) testing has rapidly expanded in endemic settings due primarily to an influx of funding from The Global Fund to Fight AIDS, Tuberculosis and Malaria, The US President's Emergency Plan for AIDS Relief (PEPFAR), the World Bank, and other donors. The most notable testing successes have been achieved in prevention of mother-to-child transmission (MTCT) programs, voluntary counseling and testing (VCT) centers, sexually transmitted disease (STD) clinics, and integrated programs pertaining to comorbidities such as malaria and tuberculosis (TB) [1]. Resource-limited settings (RLSs) oftentimes overlap with the HIV epidemic and such settings are synonymous with a lack of well-trained laboratory personnel, poor physical infrastructure, extreme climate, and geographic isolation; all of which impact the use of laboratory technologies where they are needed most.

## 2. Quality Management

Selection of point-of-care devices for HIV-endemic settings requires a quality management (QM) approach. Quality control (QC; operational approaches to ensure functional quality requirements) and quality assurance (QA; systematic approaches to ensure confidence in performance) both play a role in POC evaluation compared to a “gold standard” and implementation in the patient population. Specifically, performance characteristics such as the sensitivity (the percentage of results that are correctly identified as positive), specificity (the percentage of results that are correctly identified as negative), and robustness (performance in presence of operator, environmental conditions) should be evaluated before tests are deployed.

The sheer volume of various testing associated with HIV conducted in sub-Saharan Africa does not provide much room for testing error. A mere 0.5% error rate in 10 million tests could result in 50,000 patients misdiagnosed or mismanaged in clinical care. This error rate could lead to more transmitted infections to the unborn and sexual partners of this tested population, and could be detrimental to those requiring immediate ART. When considering a POC device for implementation in a given endemic population, it is important to validate the technique compared to a “gold standard” alternative method, specifically VL, Western blot (WB), or ELISA/EIA for HIV diagnosis, and fluorescence-activated cell sorting (FACS) for CD4 monitoring. Westgard standards [2] suggest a minimum of 40 high-quality specimens from the patient population to be compared between the POC method being evaluated and the “gold standard” laboratory technology. Larger numbers of specimens (100–200) are recommended to assess whether the new method's specificity is similar to that of the established comparator method. Specimens should be selected to cover the entire clinically significant range, as well as working range of the two methods. Bland-Altman models serve as a good measure of correlation between two methods being evaluated [2]. According to College of American Pathologists (CAP) and Clinical and Laboratory Standards Institute (CLSI) guidelines, POC technology should be compared at least twice annually to the laboratory comparator for continued performance evaluation.

## 3. HIV Testing

HIV technologies for diagnosis have included direct detection approaches based on specific viral antigen quantification such as the p24 antigen [3] and qualitative nucleic acid amplification tests (NATs) such as polymerase chain reaction (RT-PCR) amplification [4–6], and indirect or antibody-based tests such as Western blots, direct or indirect ELISA or EIA formulated into POC rapid diagnostic tests (RDTs) are widely available [7, 8]. The rapid scale-up of POC testing seen in recent times is largely attributed to successful concepts such as portable glucometers, urinalysis dipsticks, and hemoglobin spectrophotometers (e.g., HemoCue), but particularly for HIV screening using RDT [7, 8]. The bulk of these HIV RDTs are immunochromatographic tests (ICTs) performed using blood or blood derivatives, and a handful using urine [9, 10] and oral fluid [11, 12], with lateral capillary flow and quality controls incorporated into the test kit. A major shortcoming of these technologies has been poor-to-modest specificity which has promoted the use of combined RDT or combined alternative testing methods for screening and testing programs, particularly when confirming a positive diagnosis. From an immunologic aspect, RDT performance is affected by the diversity of circulating HIV subtypes (regionally as well as within an individual), HIV-2, as well as poorly understood immune cross-reactivity [13–18]. However, improvements to kit storage conditions (no need for cold-supply chain), quick turnaround time (<20 minutes), and the ease in performance make these POC tests very attractive in RLS [13–16]. RDTs are more affordable than laboratory-based tests and require no laboratory infrastructure to support scale-up. The importance of proper quality management in test evaluation, method validation, and smart implementation, as well as coordinated training requirements before use, cannot be overstressed for these POC devices to be effective.

Testing for HIV at the POC has changed the landscape of surveillance in endemic settings. The low-cost, flexible transport and storage requirements, and the lack of a need for well-trained laboratory technicians have greatly aided in their successful implementation [13–16]. With the decentralization of health care workers (HCWs) in RLS, mobility transfers and staff turn over issues, increasing visibility of counselors coupled with less-trained individuals performing RDT, adequate training, supervision and assessment will be imperative for proper sample collection, analysis, and interpretation. HCW will require initial clinician oversight, and regular structured follow-up assessment, to ensure that high-quality specimens are being collected, credible testing performed, and any training gaps correctly identified and timely addressed. Clear SOPs illustrating the stepwise technique for collection of specimens should be implemented at each site (e.g., finger-stick blood versus whole blood or interstitial fluid). Reassessment of RDT procedures will ensure high-quality blood specimens are being collected, positive patient-HCW interactions reinforced, and pain minimized. This will be very important for pediatric populations and will aid in increasing numbers tested and patient retention, if patients have a favorable view of HIV testing and community HCW.

Currently, there are seven FDA-approved HIV RDT on the market; however many more products are approved by other regulatory agencies and are currently in use [13, 19, 20]. Due to high prevalence of HIV in certain settings, employing accurate HIV RDT is not only important in patient care but also in program efficiency. As mentioned before, with a high test volume, a mere 0.5% error rate among 10 million people screened would result in 50,000 patients misdiagnosed (false-positive or false-negative).

Performance evaluation of RDT is oftentimes overlooked before implementation in endemic settings. The CDC and WHO have made great strides in supporting performance evaluation of HIV RDT in specific regions, so that Ministries of Health can roll out approved products [19, 21]. The performance characteristics of RDT in HIV-endemic settings, unfortunately, are less straightforward than what appears true with CD4 tools. Diverse HIV-1 and HIV-2 subtypes circulating regionally, as well as within patient recombinant and dual infections, all affect the performance of HIV RDT [13, 14]. Most HIV RDTs are based on antigens from HIV-1 subtype B, thus it is important to evaluate their performance based on the coating antigen and with respect to circulating subtypes [13]. One study evaluating OraQuick HIV-1/2 (OraSure Technologies, Inc., Bethlehem, PA) RDT on stored specimens from a blood bank in Kinshasa, Democratic Republic of Congo (DRC), reported 100% specificity and sensitivity among 72 known HIV-positive, non-subtype-B-confirmed specimens [22]. It will be important in the future for such controlled performance evaluations to ensue elsewhere in sub-Saharan African settings.

Studies have shown that poor performance is still encountered using approved RDT testing algorithms. Poor sensitivity and specificity has been demonstrated in controlled performance evaluations in Uganda [15, 16], the DRC [17], Ethiopia [23], and Cameroon [24]. In Uganda and the DRC, performance evaluations have demonstrated weak-positive bands with some RDTs, notably the Determine HIV 1/2 RDT (Determine; Abbott Laboratories, Germany) [15–17]. In Uganda, when weak-positive bands were included in performance evaluation, as per manufacturer's instructions, the test had low specificity (94.1%) and a low PPV (74.0%). Exclusion of the 37 samples (5.8%) with a weak-positive band improved the specificity (99.6%) and positive predictive value (97.7%) compared to EIA and Western blot (WB). In the DRC study [17], a high number of false-positive results, due to inclusion of weak-positive bands, was discovered when compared to WB and p24 EIA (Immunocomb Conbfirm HIV-1/2-antibody, Orgenics, Yavne, Israel).

 In a subsequent evaluation in the same Ugandan population [16], Determine was reevaluated, as were four new commercially available RDTs: Uni-Gold HIV (1/2) (Uni-Gold; Trinity Biotech, Ireland); STAT-PAK HIV 1/2 (STAT-PAK; Chembio Diagnostic Systems Inc., USA); Advanced Quality Rapid Anti-HIV 1/2 (Advanced Quality; IncTec Product Inc., China); First Response 1-2.0 (First Response; PMC Medical, India). All tests demonstrated a sensitivity of 100% however Determine had low specificity [85.2%, PPV 67.3% (Exact Clopper-Pearson 95% CI: 52.9, 79.7)], as did Uni-Gold and First Response [97.4%, PPV 92.1% (Exact Clopper-Pearson 95% CI: 78.6, 98.3)]. STAT-PAK performed well [specificity 99.1%, PPV 97.2% (Exact Clopper-Pearson 95% CI: 85.5, 99.9)], and Advanced Quality had sensitivity, specificity, and PPV of 100% (Exact Clopper-Pearson 95% CI: 90.0, 100.0). Including the indeterminate samples in one Cameroonian study reduced the specificity of all RDTs evaluated in this setting [24]. Genie II HIV-1/HIV-2 (Bio-Rad, Marnes-la-Coquette, France) specificity was 98.2%, and the specificities of Camstix (Camdiagnostix, Yaounde, Cameroon), Determine, and Enzygnost HIV Integral (Dade-Behring, Pennsburg, Germany) were 88.3%, 90.6%, and 92.3%, respectively. When evaluating Determine and ELISA in Ethiopia [23], these data showed 94.4% concordance in HIV antibody testing among blood donors. The sensitivity, specificity, positive and negative predictive values of Determine were 60.5%, 98.9%, 88.5%, and 94.9%, respectively.

Oral fluid sampling for HIV RDT could prove particularly useful in RLS since there would be no blood sample requirement, particularly benefiting the uptake among children. A handful of these tests have been evaluated in HIV-endemic settings. OraQuick ADVANCE Rapid HIV-1/2 (OraSure Technologies) was evaluated among 591 rural youth attending a VCT center in Zimbabwe. When compared to dry-blood spot preparations evaluated by an ELISA (Vironostika, Biomerieux BV, Boxtel, The Netherlands) and WB (MP Diagnostics, St. Ingbert, Germany) algorithm, OraQuick sensitivity and specificity were an impressive 100% [11]. In Namibia, OraQuick and OraSure Rapid HIV-1/2 (OraSure Technologies) RDTs were evaluated among pregnant women, who provided oral fluid samples and paired blood samples for dual ELISA comparison [AxSYM HIV 1/2 gO (Abbott, Abbott Park, IL); Access HIV 1/2 New test (Bio-Rad Laboratories)]. OraQuick results for 273 women revealed 100% specificity and sensitivity, whereas OraSure results from the same women yielded 97.1% sensitivity and 99.5% specificity. Concordance between OraQuick and OraSure results was high ([kappa] = 0.97; 95% CI: 0.95–0.99) [12]. Continued performance evaluations of oral fluid specimens for RDT are greatly needed, particularly in young infants and children, pregnant and lactating mothers, as well as in various HIV-subtype settings to account for potential immunologic variation.

Fourth-generation HIV RDTs are being developed and warrant extensive performance evaluation in the field. One such test, ARCHITECT HIV Ag/Ab Combo Assay (Abbott Laboratories), was FDA-approved in 2010 as the first RDT that detects antigen and antibodies for HIV simultaneously [20]. In addition, it has been indicated for use in pregnant women and children ≥2 years of age and in diagnosing acute HIV-1 infection. The test incorporates the HIV-1 p24 antigen as well as antibodies to HIV-1 groups M and O, as well as antibodies to HIV-2. The Determine HIV 1/2 Ag/Ab Combo (Inverness BioMedical, Waltham, MA) was recently evaluated by the CDC/USAID and provided HIV antigen detection about 10 days (mean time) before antibody detection when using seroconversion panels in a controlled laboratory setting [13]; however this promising RDT will need to be evaluated in diverse field settings and in areas of various circulating subtypes and background pathologies. The potential benefit of including such an RDT in HIV screening programs could be useful in detecting recent infections, prior to the emergence of HIV antibodies, therefore reducing the window period of antibody detection. The median detection time was demonstrated to be 7 days earlier (range 0 to 20 days) compared to 3rd-generation EIA tests to which they were compared [20]. 

External quality assessment (EQA) programs for HIV RDT are greatly needed in sub-Saharan Africa in order to ensure selected tests are performing accurately and according to manufacturer's expectations. The CAP provides member laboratories proficiency testing (PT) panels for anti-HIV-1/2 as well as HIV-1 p24 for peer comparison. Programs modeled after the CAP PT program will enable regional EQA programs to improve the performance of selected tests and improve testing accuracy.

In the aforemenioned studies in sub-Saharan Africa, RDT sensitivities ranged from 94.1% to 100%, and specificities ranged from 85.2% to 100% when performed and interpreted according manufacturers' instructions [9, 11, 12, 15–17, 23, 24]. HIV EQA programs, targeted specifically at ICT RDT, will likely improve the performance of selected tests and improve diagnostic accuracy overall in sub-Saharan Africa. Misdiagnoses of HIV infection can cause improper patient care and allow for progression to AIDS if not identified early, thus these studies suggest a three-part QM approach to implementing HIV RDT. First, validation of a RDT in a specific population must be conducted in comparison to known local HIV-positive and HIV-negative panels with confirmatory testing by WB or EIA. It is possible that HIV-subtype immune responses may affect the reactivity on RDT [14–17, 23]. Secondly, there is a need to confirm indeterminate bands on RDT by alternative methods before a result is determined and a diagnosis is assigned. Given the social and psychological consequences of a false-positive HIV diagnosis, as well as the probably poor clinical outcomes and onward HIV transmissions among those given a false-negative result, confirmatory testing is needed. To increase accuracy of RDT, a multiple-test algorithm, specific to the population, should be validated to account for subtype-specific immune responses and nonspecific immune cross-reactivity of antibodies. When implementing HIV RDT testing algorithms involving multiple RDTs, countries need to provide clear guidance on how to deal with discordant results based on the specific findings of performance evaluations.

Fourth-generation tests potentially could shorten the window period of a positive antibody result, thus detecting infections earlier and preventing onward transmissions. However, due to the relatively new arrival of these tests from the regulatory pipeline, performance evaluations in various RLSs, and among diverse patient populations (HIV subtype distribution, various ages, varying VL, pregnant) is warranted before implementation. Nonblood specimen platforms, such as those based on urine and oral fluid, should be evaluated to increase the uptake, particularly in remote settings where turnaround times are long, and it may be unfeasible to wait and return for ELISA/EIA results. POC results distribution at the time of testing could provide ample opportunity for risk reduction counseling and decrease long-term followup among these patients.

In sub-Saharan Africa, the cost of an HIV RDT ranges from $1.23 to $4.00 per test [13]. The small kit size, light weight, as well as minimal separate reagent components, enable bulk shipment of these RDTs from distributors and eventual onward transport by post or road vehicle to remote sites.

## 4. HIV Clinical Monitoring

In fiscal year 2010, nearly 33 million people were tested for HIV solely in PEPFAR countries, an enormous scale up from previous years [25]. With HIV testing at an all-time high and new “test and treat” programs being explored [26], this will result in millions more positive diagnoses and will require clinical laboratory monitoring for those who are eligible for antiretroviral therapy (ART). Clinicians are gradually moving away from using clinical presentation and/or total lymphocyte counts to determine ART eligibility; however, many ART programs in RLS still rely on immunologic and/or clinical presentation to measure response to therapy and to determine when to change to a second-line regimen [27–30]. The 2010 WHO/UNAIDS recommendations call for all those who are diagnosed HIV-positive to have access to CD4 counts and use an ART treatment cut-off of ≤350 CD4+ T-cells/*μ*L for adults and adolescents [31]. The CDC has established a treatment cut-off CD4+ T-cell percentage of <25% for infants under 11 months of age, <20% for children up to 3 years of age, and <15% for children between 3 and 5 years of age [32].

CD4 counts are primarily determined by FACS on flow cytometers which are costly, require a cold chain, need routine technical maintenance, require skilled technicians, and are primarily located in urban centers. When considered alone, CD4 data have been shown to misclassify patients as immunologic ART failures in Malawi (30% misclassified) [27], Uganda (20.2% misclassified) [29], and South Africa (18.5% misclassified) [28]. Relying solely on clinical presentation for ART initiation can result in poor patient care and can lead to AIDS progression or death. Clinical presentation had a low sensitivity of 15.2% in determining ART eligibility in South Africa [28] and resulted in 57% of patients being misclassified in Malawi [27]. It also has correlated with death within 30 months among 41.3% of patients followed in Uganda [29].

Efforts are underway to bring the clinical laboratory to the patient for clinical care. POC devices for CD4 immunologic monitoring [33–35] and toxicity monitoring (e.g., lactate, renal function tests [RFT], liver function tests [LFT]) [36, 37] are currently being evaluated or in the development pipeline. These tools have the potential to change the landscape of clinical care among HIV-infected patients in RLS, but only packaged with proper training and good quality management programs will we be able to realize their utility.

Clinical monitoring of HIV disease by CD4 enumeration would greatly be enhanced with POC devices. If validated, these devices could rapidly and accurately identify CD4 counts with minimal operator training, infrastructural setup and with less cost than standard laboratory-based equipment such as flow cytometers. These machines have a quick turnaround time per test (5–30 minutes), are fully automated with minimal sample volume, minimal preanalytical sample processing, and no cold chain requirements. Additionally, flexible power supply options to allow usage in RLS, combined reagents, and minimal supplies, simplify supply-chain management. They are also usually enabled to provide instant paper results for attachment to patients documents as well as inbuilt data storage units. The benefit of POC machines in patient care lies greatly in shortening the decision time pending results from conventional machines, resulting in real-time treatment decisions, since patients routinely walk or commute for great distances to reach ART treatment programs in RLS.

It is important to note that CD4 indices are crucial for monitoring pre-ART disease progression but may perform suboptimally in identifying treatment failure as compared to VL quantification [27–30] but given the expense and logistical issues associated with NAT platforms, CD4 evaluation even on ART treatment provides a fairly reasonable monitoring option in RLS. One promising POC monitoring device is the PIMA analyzer (Alere, Inc., Waltham, MA) which enumerates CD4 counts in 20 min from a finger-stick blood sample or blood in EDTA. The PIMA has been evaluated in South Africa and in Zimbabwe and performs well compared to gold standard FACS analysis. In a South African mobile VCT clinic setting [35], the PIMA was evaluated on venous and finger-stick blood across three different PIMA devices in parallel, and with four different technicians. Performance was adequate compared to EPICS XL-MCL cytometry (Beckman Coulter) on venous blood [*R* ^2^ = 0.92, mean difference (Bland Altman) = –12 cells/*μ*L, (95% CI –23 to –1), 94% sensitivity, 98% specificity, 84% PPV]. Using finger-stick blood, the PIMA also performed well [*R* ^2^ = 0.92, mean difference = 15 cells/*μ*L, (95% CI –9 to 39), 100% sensitivity, 98% specificity, 67% PPV]. In Zimbabwe, finger-stick blood with a PIMA performed well when compared to venous blood on a FACSCalibur (Becton Dickinson) [mean absolute CD4 difference = +7.6 cells/*μ*L, *P* = 0.72] [33]. In the same study, PIMA and FACSCalibur analyses conducted by both nurses [mean absolute CD4 = +18.0 cells/*μ*L, *P* = 0.49] and laboratory technicians [mean absolute CD4 = −3.1 cells/*μ*L, *P* = 0.93] indicated adequate performance which provides access to larger manpower in RLS.

Becton Dickinson is developing an easy-to-use, image-based counting technology suitable for RLS that will provide CD4 absolute count, CD4%, and hemoglobin, all on the same single-use disposable cartridge. Features of the automated device will include touch screen user interface, flexible workflow with high throughput, an integrated microprinter, battery or solar-powered capability, and data archive/transfer capabilities. The sample is collected from the patient using a finger-stick or from an EDTA tube. After a short incubation period the cartridge is read quickly requiring only a single step. The new and innovative cartridge technology contains dried reagents with no cold chain requirements, which enables longer shelf life over a wide range of environmental conditions (Becton Dickinson, Mikulski L., personal communication, 2011).

It will be important for continued performance evaluations of PIMA and other devices, with consideration for robustness measures, such as evaluation in remote setting that are hot and either humid or dry, among large numbers of young infants and children with finger-prick sampling, as well as over a broad range of CD4 counts in HIV-positive patients. Performance evaluation among lay counselors, such as in VCT centers, should be also be pursued. Also important to consider will be the cost of such instruments and associated consumables as many ART programs have financial constraints due to the sheer number of patients in their care.

When implementing POC tests for CD4 enumeration, EQA is an important aspect of the total QMS. EQA bodies such as the United Kingdon Natioanl Quality Assessment Service (UKNEQAS) and CAP implement programs for member laboratories which perform CD4 enumerations. Proficiency testing panels are distributed six times per annum for UKNEQAS and three times per annum for CAP, and member laboratories are graded on their accuracy compared to known specimens.

HIV-positive patients on ART, particularly stavudine regimens, are at risk for systemic toxicity, including lactic acidosis, renal and/or kidney function impairment, and even death [32, 36, 37]. Clinical chemistry laboratory service is oftentimes limited to hospitals and ART centers in urban areas, is of questionable quality, and can have long turnaround times for results delivery. Several POC devices have been evaluated in sub-Saharan Africa and elsewhere for performance and utility. At two clinics in Uganda, the Accutrend Lactate analyzer (Accutrend, Roche Diagnostics, Mannheim, Germany) was accurate, reliable, and cost effective [35–37]. In one study [36], there was good correlation comparing Accutrend to the Cobas Integra 400 plus (Roche) hospital standard method [*R* ^2^ = 0.94, bias = −0.06], demonstrating superb agreement between the POC instrument and the laboratory method. In another Ugandan study [37], the Accutrend determined lactate of ≥4.0 mmol/L was 88.3% sensitive and 71.2% specific at determining in-hospital mortality among a predominantly HIV-infected severe sepsis cohort. Overall, a 7-fold increase in mortality was determined at a cut-off lactate measurement of 4.0 mmol/L indicating that accurate, reliable testing is imperative for proper patient care in this population.

In a recent study in Mozambique [34], nurses performed POC clinical chemistry with the Reflotron Plus (Roche) for alanine aminotransferase (ALT) and aspartate aminotransferase (AST) on finger-stick blood. Tests for ALT on the Reflotron device had a mean bias of −0.2 U/l (LOA −10.5 to 10.0) whereas AST testing had a bias of −4.0 U/l (LOA −44.5 to 36.5). These results were similar to those observed when the POC testing was performed by laboratory technicians, resulting in a bias of −4.8 U/l (LOA −35.6 to 25.9) for ALT and −6.4 U/l (LOA −29.8 to 17.0) for AST.

## 5. Diagnosing Comorbidities

In many settings, there is a need for RDT to diagnose co-morbidities such as malaria and TB to guide clinical care in HIV-endemic areas [38–42]. In sub-Saharan Africa, presumptive treatment of fevers with malaria drugs is extremely common, and laboratory-confirmed malaria diagnoses are not uniformly conducted. As with any ICT RDT, test kits should be stored and transported according to properly listed conditions in the package insert, should be performed according to manufacturer's SOP for individual assays in a well-lit setting, and should be read for interpretation by personnel trained on the specific test. Development of positive control bands is used to judge validity with confirmatory testing either by “gold standard” smear microscopy or a second RDT. If two sequential tests do not demonstrate control bands, tests should be considered invalid, and a blood smear read. 

Very few studies have evaluated RDT for malaria diagnosis in HIV-positive populations. The Binax Now Malaria RDT (Binax; Inverness Medical Innovations, Inc., Waltham, MA) is an ICT based on the pan-Plasmodium antigen, histidine-rich protein-2 (HRP-2) and has been evaluated in rural mobile clinics in Uganda by clinicians evaluating febrile adult HIV patients [38]. Compared to laboratory-confirmed thick blood smears, Binax sensitivity was 85.7% (95% CI: 57.2–98.2) and specificity was 97.8% (95% CI: 94.9–99.3) indicating favorable, although imperfect performance, for excluding malaria as the cause of fevers among HIV-positive febrile patients. In another study in the same Ugandan population [39], Binax demonstrated a malaria prevalence of 31.1% among screened mothers, but no significant difference was observed between HIV-positive mothers compared with HIV-negative mothers (30.3% versus 32.3%, *P* = 0.72). Vertical HIV transmission was significantly higher if mothers had a positive Binax test (RR = 3.2, 95% CI: 1.14 to 9.2). The highest rates of MTCT were among mothers who had both placental malaria infection and Binax-diagnosed malaria (30.4%), indicating the importance of using malaria RDT, particularly in PMTCT programs [39].

Increasing TB drug resistance, coupled with a growing number of patients coinfected with TB and HIV, has highlighted the urgent need for more accurate and RDT, with a POC device being an ultimate goal. Many patients in sub-Saharan Africa have limited access to laboratory testing which contributes to high rates of death during co-infection. The Foundation for Innovative and Novel Diagnostics (FIND, Geneva, Switzerland) is working with industry and regulatory partners, to develop and validate RDT products in TB, as well as in other disease areas. FIND has determined that a successful POC test will consist of one which is used where patients seek medical care, since many times, diagnosis and treatment are based on clinical symptoms [41]. Current platforms being evaluated include urinary antigen detection and antibody detection at the POC.

The pan-mycobacterial antigen lipoarabinomannan (LAM) has been identified as a promising candidate to incorporate into a POC test because of its temperature stability and detection in urine [42]. With hopes to build upon commercially available ELISA platforms for the creation of lateral flow platform RDT, two studies [20, 43] have been conducted to detect LAM in urine specimens with ELISA. In Zimbabwe [43], the LAM ELISA sensitivity was 44% (95% CI 36–52) for culture-confirmed TB (52% in smear-positive patients) and specificity was 89% (95% CI 81–94). Sensitivity was significantly higher in HIV- and TB- coinfected patients (52%, 95% CI 43–62, *P* < 0.001) compared to HIV-negative TB patients (21%, 95% CI 9–37). Sensitivity in smear-negative patients was very low (28%, 95% CI 13–43) for combined HIV-positive and HIV-negative patients. In a Tanzanian study [42], of all patients which had a positive sputum culture, LAM ELISA was 80.3% sensitive. Sensitivity of acid-fast bacilli sputum microscopy was 62.1%. TB was diagnosed in 195 (49%), including 161 culture-positive patients and excluded in 114 (29%) participants. LAM ELISA sensitivity was 44% (95% CI 36–52) for culture-confirmed TB (52% in smear-positive patients) and specificity was 89% (95% CI 81–94).

Despite these imperfect study results, the search for a sensitive platform to detect TB antigen or antibody responses continues. FIND has partnered with Antigen Discovery Inc. and the Public Health Research Institute (USA) to dissect the *Mycobacterium tuberculosis* proteome, in a search towards identifying a panel of diagnostically relevant antigens for TB serology [40, 43]. The TB proteome arrays are being screened with TB patient sera and controls from across the globe. Patient cohorts include those with and without HIV infection and those with latent tuberculosis. FIND reports that a limited number of combined antigen targets may be translated into an RDT for active TB detection. Numerous technological platforms appropriate for a broad range of analyses have been evaluated, and a first prototype assay is expected in 2011 [40]. It will be important for such assays to be evaluated across robust conditions with consideration for test operators, climate, immune status, varied TB pathologic states, and co-infections.

## 6. Conclusion

In order to achieve the ambitious goals of “test and treat” program implementation and to promote universal access of HIV-positive patients to ART in RLS, we must decentralize laboratory technology for screening and clinical monitoring. POC devices have proven to be easy to transport, operate, and maintain in RLS [7–9, 11, 44]. Additionally, lower skilled staff, such as lay counselors and nurses, are equally able to perform these POC tests compared to trained laboratory technicians [8, 9, 12, 14, 16, 36, 38]. Access to this additional human resource will provide a great advantage to avail testing and monitoring in the field, where typically, health care staff are less trained and less technically savvy.

Another key to the successful implementation of POC devices will be cost. Current laboratory-based testing and monitoring technologies may be too expensive for most programs in RLS, particularly for ART programs. In sub-Saharan Africa, central laboratories come potentially with a greater expense due to human resource and infrastructural requirements. Only when combined with good QM systems to ensure accuracy will these POC devices really transform the availability of tests in real time to inform proper patient care.

Numerous HIV RDTs are available, and many are being evaluated in RLS with support from the US CDC and others [13, 14, 16, 18, 19, 21]. HIV RDTs have great potential when evaluated in strategic QM performance evaluations, spanning various VL concentrations, HIV disease states and when considering circulating subtypes. Consistent EQA and retraining, particularly of lay persons, will be needed to sustain their accuracy and availability in RLS. It is of utmost importance that personnel are trained in proper sample collection, with clear SOPs for various collection procedures and biospecimen types to ensure a quality specimen. The PIMA POC CD4 instrument is currently available in sub-Saharan Africa and has handful of promising studies validating its use in RLS among HIV-positive patients to monitor ART response [34, 35]. A POC VL device is greatly needed to contribute to clinical decision making, particularly in regions which are far remote and where financial burden prohibits routine VL to monitor ART. To accompany HIV RDT, malaria and TB RDTs have the potential to add to the diagnostic capacity in RLS and improve patient care in areas of overlapping endemicity [38, 39, 41, 42]. Collaborative efforts have the capacity to combine technical expertise, regulatory capacity as well as implementation expertise for speeding up the development pipeline of products, performance evaluation, and implementation at the country and community level [13, 18, 19, 40].

##  Conflict of Interests 

The authors report no conflict of interest.
